# When Local Beats Systemic: Durable Control of Metastatic Undifferentiated Uterine Sarcoma and Surveillance Uncovering a Second Primary

**DOI:** 10.15388/Amed.2026.33.1.18

**Published:** 2026-07-22

**Authors:** Ignas Lapeikis, Vakarė Baranauskytė, Dainius Jančiauskas, Lina Pužauskienė, Erika Korobeinikova

**Affiliations:** 1 Lithuanian University of Health Sciences, Faculty of Medicine, Kaunas, Lithuania; 2 Department of Pathological Anatomy, Lithuanian University of Health Sciences, Kaunas, Lithuania; 3 Oncology Institute of Lithuanian University of Health Sciences, Kaunas, Lithuania

**Keywords:** undifferentiated uterine sarcoma, pulmonary metastases, oligometastatic disease, invasive ductal carcinoma, stereotactic radiotherapy, secondary primary malignancy, nediferencijuota gimdos sarkoma, plaučių metastazės, oligometastazinė liga, invazinė duktalinė karcinoma, stereotaksinė spindulinė terapija, antrasis pirminis piktybinis navikas

## Abstract

**Introduction:**

Undifferentiated uterine sarcoma (UUS) is a rare, aggressive uterine mesenchymal malignancy with poor prognosis, for which, evidence supporting metastasis-directed treatment strategies is limited.

**Case presentation:**

A 59-year-old woman underwent total hysterectomy with bilateral salpingo-oophorectomy (R0) for stage IB UUS in 2016, followed by adjuvant doxorubicin–ifosfamide. Three years later, surveillance CT identified bilateral pulmonary metastases. Video-assisted thoracoscopic resection confirmed metastatic UUS, and stereotactic body radiotherapy (SBRT) was delivered to residual and subsequent lung lesions, achieving sustained local control without systemic therapy. The patient remained progression-free for almost three years. In 2024, follow-up imaging showed no active sarcoma but incidentally detected a left breast lesion; biopsy revealed invasive ductal carcinoma with mucinous features. She underwent breast-conserving surgery with sentinel lymph node biopsy (pT1N0(sn)), followed by adjuvant whole-breast radiotherapy and endocrine therapy with tamoxifen.

**Outcomes:**

At 8.5 years from UUS diagnosis and one year after breast cancer treatment, the patient remains alive with no evidence of recurrent or metastatic disease and with excellent performance status (ECOG 0).

**Conclusions:**

This case demonstrates prolonged, chemotherapy-free control of metastatic UUS using a metastasis-directed strategy combining surgery and SBRT, and highlights the importance of continued surveillance for secondary primary malignancies. It supports considering an oligometastatic treatment paradigm and multidisciplinary management in selected UUS patients despite the absence of prospective data.

## Abstract (it)

**Įvadas:**

Nediferencijuota gimdos sarkoma (angl. UUS) yra reta, agresyvi gimdos mezenchiminė piktybinė liga, pasižyminti bloga prognoze, o šio naviko metastazėms skiriamo gydymo strategijų veiksmingumą pagrindžiantys duomenys yra riboti.

**Klinikinis atvejis:**

59 metų pacientei 2016 m. dėl IB stadijos UUS atlikta totalinė histerektomija su abipuse salpingooforektomija (R0), po kurios taikyta adjuvantinė doksorubicino ir ifosfamido chemoterapija. Po trejų metų stebėjimo atlikus kompiuterinę tomografiją pacientei nustatytos abipusės naviko metastazės plaučiuose. Vaizdu asistuojamosios torakoskopinės operacijos metu jos buvo pašalintos, histologiškai patvirtinta metastazavusi UUS. Vėliau likusiems ir naujai atsiradusiems plaučių židiniams taikyta stereotaksinė kūno spindulinė terapija (angl. SBRT), pasiekta ilgalaikė lokali kontrolė be sisteminio gydymo. Liga neprogresavo beveik trejus metus. 2024 m. kontrolinių tyrimų metu aktyvios sarkomos požymių nenustatyta, tačiau atsitiktinai aptiktas kairės krūties darinys; biopsija patvirtino invazinę latakinę karcinomą su mucininiais požymiais. Atlikta krūtį tausojanti operacija su sarginio limfmazgio biopsija (pT1N0(sn)), po kurios skirta adjuvantinė visos krūties spindulinė terapija ir endokrininis gydymas tamoksifenu.

**Rezultatai:**

Praėjus 8,5 metų nuo UUS diagnozės ir vieneriems metams po krūties vėžio gydymo pacientė yra gyva, be ligos recidyvo ar metastazių požymių, išlieka puiki funkcinė būklė (ECOG 0).

**Išvados:**

Šis klinikinis atvejis parodo, kokia svarbi yra ilgalaikė metastazavusios UUS kontrolė be chemoterapijos, taikant į metastazes nukreiptą strategiją, kurios tikslas lokaliomis priemonėmis sunaikinti konkrečius vėžio židinius (metastazes), derinant chirurginį gydymą ir SBRT, bei pabrėžia tęstinės stebėsenos svarbą antrinių pirminių navikų diagnostikai. Gauti duomenys leidžia svarstyti oligometastazinės ligos gydymo paradigmą ir daugiadalykės komandos vaidmenį atrinktiems UUS pacientams, nepaisant prospektyvių tyrimų stokos.

## Introduction

*Undifferentiated Uterine Sarcoma* (UUS) is defined by the World Health Organization as a rare, high-grade mesenchymal malignancy of the uterus, lacking evidence of specific lineage of differentiation [1]. UUS accounts for less than 1% of all uterine cancers and approximately 5–7% of uterine sarcomas [2]. It typically presents in women between the ages of 40 and 70, most often with abnormal uterine bleeding, pelvic pain, or a rapidly enlarging uterine mass [3]. These sarcomas are often detected at advanced stages, which is consistent with their inherently aggressive biology [4].

The prognosis of UUS is extremely poor. The median overall survival is reported to be less than two years, and five-year survival in patients with advanced or metastatic disease ranges between 10% and 25% [4,5]. Hematogenous dissemination is common, with the lungs being the most frequent site of distant metastasis, followed by peritoneum and bone [3,6].

The recommended standard treatment for UUS is radical surgery, typically total hysterectomy with bilateral salpingo-oophorectomy [2,7]. However, most patients are considered for adjuvant systemic therapy due to the high risk of relapse, with anthracycline-based regimens most commonly employed. In the metastatic setting, doxorubicin with or without ifosfamide remains the standard first-line treatment, while gemcitabine–docetaxel is regarded as an alternative option [8,9]. Nevertheless, systemic therapy results in limited responses, and achieving durable disease control remains challenging [7,8].

In the oligometastatic setting, local ablative approaches have gained increasing attention. Stereotactic body radiotherapy (SBRT) has shown encouraging results in controlling pulmonary and other sarcoma metastases, with reported local control rates of up to 80% at two years [10]. Although prospective data specific to UUS are lacking, SBRT appears to provide a safe and effective option for selected patients [10,11].

Durable survival in UUS is uncommon, and management is challenged not only by the risk of recurrence but also by the increased incidence of second primary malignancies and their impact on health-related quality of life [12,13]. These data underscore the importance of comprehensive, long-term follow-up strategies that combine oncological surveillance for both recurrence and second primaries with supportive care interventions aimed at maintaining function and quality of life.

Here, we present a rare and clinically instructive case of recurrent metastatic UUS with pulmonary metastases, managed successfully with multimodal therapy including adjuvant systemic chemotherapy and repeated SBRT. Remarkably, the patient achieved prolonged survival of more than eight years and was subsequently diagnosed with a second primary invasive ductal breast carcinoma, emphasizing the importance of individualized long-term surveillance strategies.

## Case Presentation

**Figure 1:**
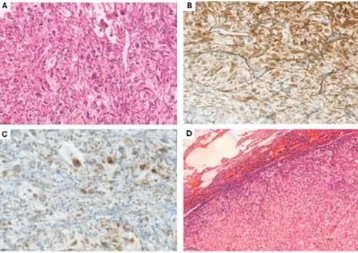
Microscopic findings. Haematoxylin and eosin staining of the primary uterine tumour demonstrated a highly cellular neoplasm composed of polymorphic cells arranged in disorganized fascicles and solid nests within focal myxoid stroma. Areas of tumour necrosis, marked nuclear atypia, and frequent mitotic figures were evident (A). Immunohistochemistry showed focal positivity for vimentin (B) and CD10 (C), with scattered reactivity for cytokeratin and CK18, while other markers were negative (see also Table 1). Histology of a resected pulmonary nodule confirmed metastatic undifferentiated uterine sarcoma with morphology identical to the primary tumour (D).

59-year-old woman was diagnosed undifferentiated uterine sarcoma in 2016. A total hysterectomy with bilateral salpingo-oophorectomy was performed *en bloc*, without morcellation. Histopathological examination revealed a uterine corpus tumour exceeding 5 cm, composed of polymorphic cells arranged in disorganized fascicles and nests within focal myxoid stroma (see Figure 1). The mitotic index was not assessed, despite marked nuclear pleomorphism. Tumour necrosis was present, no lymphovascular invasion was identified. The adnexa, adipose tissue, and regional lymph nodes were free of tumour infiltration. Immunohistochemistry showed negative hormonal receptors (ER-, PR-) and focal vimentin and CD10 positivity, scattered cytokeratin and CK18 reactivity, and negativity for smooth muscle, myogenic, and neural markers (see Table 1). According to the TNM (FIGO 2009) classification, the tumour was staged as pT1bN0M0, stage IB. After discussion in the multidisciplinary team, considering the patient’s age, good performance status, the aggressive nature of the tumour and the high risk of recurrence, adjuvant chemotherapy was initiated, consisting of four cycles of doxorubicin and ifosfamide.

**Table 1:** Immunohistochemical profile of the uterine tumour

Marker	Result	Pattern/Comment
Vimentin (VIM)	Positive (focal)	Cytoplasmic staining in tumour cells
CD10	Positive (focal)	Cytoplasmic/membranous staining
Cytokeratin (CK)	Positive (scattered)	Few scattered tumour cells positive
CK18	Positive (scattered)	Weak cytoplasmic staining
SMA (Actin)	Negative	No cytoplasmic staining detected
Desmin	Negative	No cytoplasmic staining detected
MyoD1	Negative	No nuclear staining
S100	Negative	No cytoplasmic/nuclear staining
CK5	Negative	No staining detected
CK7	Negative	No staining detected

**Figure 2:**
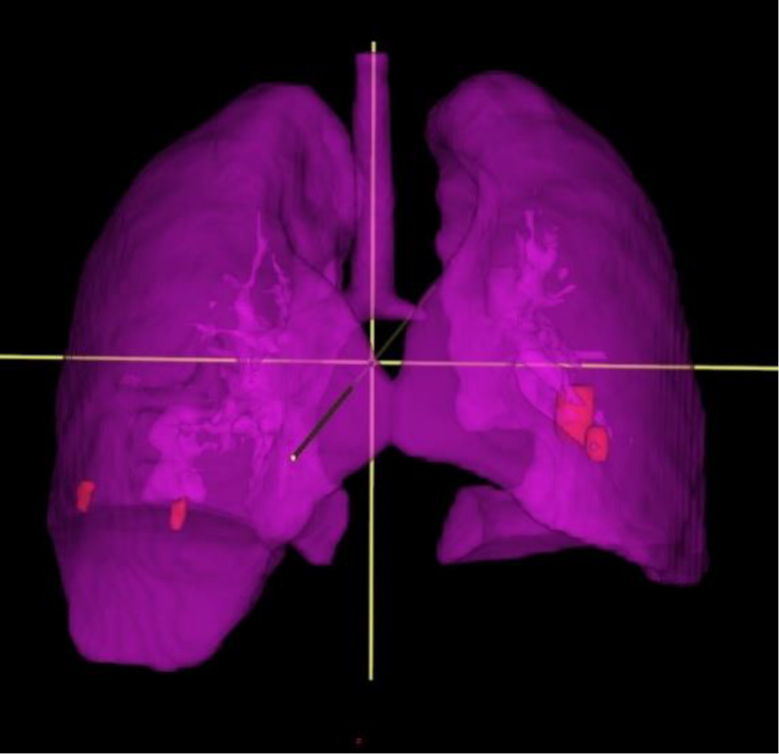
Three-dimensional chest CT reconstruction demonstrating multiple bilateral pulmonary metastases from undifferentiated uterine sarcoma.

In September 2019, chest CT revealed six bilateral pulmonary nodules, ranging from 0.5 to 1.5 cm in diameter, distributed across segments S4/S5, S9, S5, and S8/6, including subpleural locations. PET/CT confirmed low-grade FDG uptake confined to those bilateral pulmonary nodules, with no metabolic evidence of disease in other locations. Given the patient’s history of papillary thyroid carcinoma treated in 2009, metastatic disease of thyroid origin was considered in the differential diagnosis. Following video-assisted thoracoscopic right atypical resection (VATS) was performed. Histopathological examination confirmed metastasis of the primary undifferentiated uterine sarcoma. The patient underwent stereotactic body radiotherapy (SBRT) directed to four lesions not resected at surgery with a total dose of 45 Gy delivered in 5 fractions (see Figure 2 and Table 2).

**Table 2:** Radiotherapy treatment planning parameters for stereotactic radiotherapy (SRT) of pulmonary metastases. Planning was performed on a Varian TrueBeam system using VMAT (volumetric modulated arc therapy) with 10 MV flattening-filter-free (FFF) beams

Field ID	Technique	Machine/Energy	MLC	Gantry angle (°)	Collimator angle (°)	Couch angle (°)	Field size (X × Y, cm)	SSD (cm)	MU	Ref. Dose (Gy)
000p	Static-I	TRUEBEAM01 – 6X	–	0	0	0	10.0 × 10.0	90.0	–	–
000→179	SRS Arc Therapy-I	TRUEBEAM01 – 10X-FFF	VMAT	0 → 179 (CW)	30	0	5.1 × 4.4	90.0	2975.4	–
179→000	SRS Arc Therapy-I	TRUEBEAM01 – 10X-FFF	VMAT	179 → 0 (CCW)	330	0	5.1 × 4.3	91.7	3166.6	–
Gantry_0	Static-I	TRUEBEAM01 – 6X	–	0	0	0	10.0 × 10.0	90.0	–	–
270p	Static-I	TRUEBEAM01 – 6X	–	270	0	0	10.0 × 10.0	74.8	–	–

MU = monitor units; SSD = source-to-surface distance; MLC = multileaf collimator; CW = clockwise; CCW = counterclockwise

**Figure 3:**
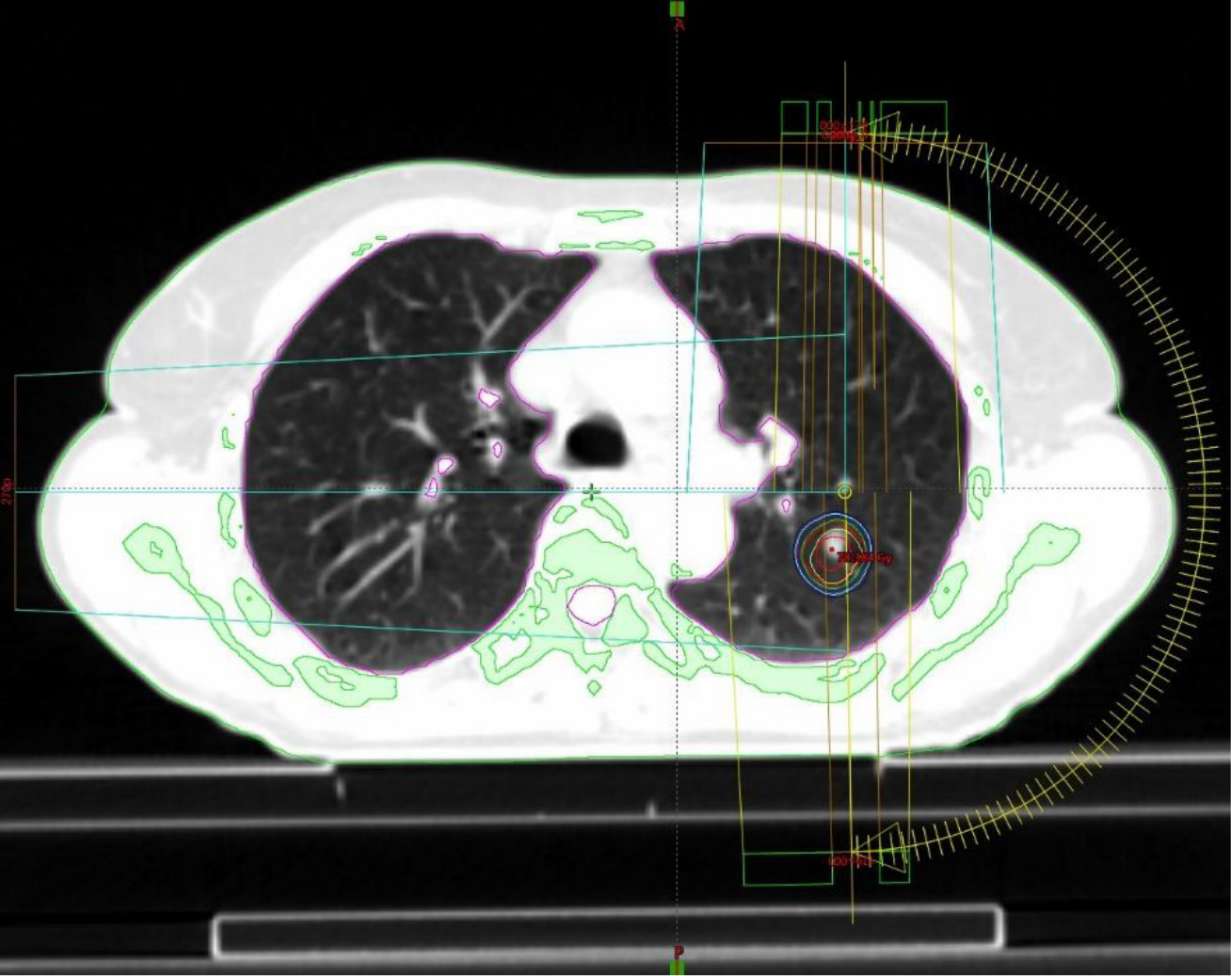
Stereotactic body radiotherapy planning for pulmonary metastasis. Treatment planning computed tomography scan of the thorax demonstrating a metastatic nodule in the right lung (target volume marked with concentric isodose lines). Dose distribution was achieved using volumetric modulated arc therapy (VMAT)-based stereotactic body radiotherapy, delivering 60 Gy in three fractions with precise target coverage and sparing of the surrounding normal lung tissue.

At the subsequent follow-up in 2020, PET/CT demonstrated fibrotic changes in the previously treated lesions but revealed two new pulmonary nodules, only one of which showed FDG uptake and was classified as metastatic. SBRT (60 Gy in 3 fractions) again was administered to a lesion (see Figure 3) in the left lung (segment S3). Follow-up chest and abdominal imaging over almost three years showed no signs of disease progression.

In 2021, radiological imaging showed no pathological pulmonary lesions, but incidental breast mass was detected. After subsequent assessment, the patient was diagnosed with an invasive ductal carcinoma without lymph node spread and distant metastasis. Lumpectomy with sentinel lymph node biopsy was done. Histology confirmed invasive ductal carcinoma with a mucinous carcinoma component pT1N0(sn) LVI1 R0 G2 luminal B subtype (ER+, PR-, HER2 (0), Ki-67 30%). She subsequently received radiotherapy to the left breast, delivering 40.05 Gy in 15 fractions. Endocrine therapy with Tamoxifen was initiated thereafter.

The patient currently remains under active surveillance. At last follow-up, nearly one year after breast cancer treatment and 8.5 years after the initial diagnosis of uterine sarcoma, the patient is alive with no radiologic evidence of disease progression. This case highlights durable survival in metastatic undifferentiated uterine sarcoma attained through local therapeutic approaches alone, with no systemic treatment administered after recurrence.

## Discussion

This case presents a striking paradox: an undifferentiated uterine sarcoma with highly aggressive histologic features and early metastatic recurrence, yet an exceptionally long survival of 8.5 years, achieved through local treatments only (VATS and SBRT) without sustained systemic therapy after recurrence. Although the tumour was ER–/PR–, excluding a hormonally favourable subtype, the marked nuclear pleomorphism was more consistent with mitotic dysregulation and genomic instability than with high proliferative activity. This biological profile may have contributed to the unusually indolent oligometastatic course and prolonged disease control observed. In contrast, most series report a median overall survival of less than 2 years for patients with undifferentiated uterine sarcoma, even with multimodal treatment [14–16]. This outcome therefore challenges prevailing assumptions regarding the natural history and therapeutic responsiveness of this rare malignancy.

### Histopathological and molecular considerations

Undifferentiated uterine sarcoma (UUS) remains a diagnosis of exclusion, established only after careful evaluation has ruled out other defined uterine sarcoma subtypes. By definition, UUS is a high-grade malignancy, lacking specific differentiation and exhibiting highly aggressive biological behaviour. In clinical practice, uterine sarcomas can usually be distinguished morphologically and confirmed by immunohistochemistry (IHC). Most common are leiomyosarcomas, recognized by spindle cell fascicles with smooth-muscle marker expression. Low-grade endometrial stromal sarcomas by monotonous stromal cells infiltrating the myometrium and diffusely expressing CD10 and ER/PR. Adenosarcomas show a biphasic epithelial–stromal pattern with stromal overgrowth and atypia, which are features that collectively distinguish these entities from UUS. By contrast, UUS lacks any recognizable line of differentiation, instead showing marked pleomorphism, brisk mitotic activity, necrosis, and destructive myometrial invasion. Immunohistochemically, UUS often exhibits aberrant p53 and diffuse p16 expression, while ER/PR and CD10 are variable and usually only focal. Therefore, with the characteristic histopathologic features of the other uterine sarcomas established, the key question in the differential diagnosis is the distinction between UUS and HG ESS, which, although also high-grade, typically retains a more uniform stromal-like appearance. When morphology and IHC remain inconclusive, molecular testing (FISH, PCR or NGS) – can refine classification: HG ESS is defined by recurrent genetic fusions such as YWHAE–NUTM2 or BCOR rearrangements, with corresponding cyclin D1 or BCOR protein overexpression [17-19]. In contrast, UUS lacks consistent recurrent alterations, and integrated molecular analyses suggest that it represents a heterogeneous group of tumours [20]. Prognostic classification models incorporating mitotic index, hormone receptor status, and molecular surrogates are being developed [21]. For this reason, expert pathology review and immunohistochemical confirmation are essential to avoid misclassification, which directly affects systemic treatment strategies.

### Adjuvant chemotherapy in early-stage uterine sarcomas

Adjuvant therapy in stage I uterine sarcomas remains highly controversial as randomized evidence is limited and inconclusive. The EORTC 55874 trial demonstrated that pelvic radiotherapy improved local control but had no effect on survival in stage I–II disease [22], and most relapses were distant, predominantly in the lungs, and thus cannot be affected by local therapy; therefore, radiotherapy after surgery is not routinely recommended. Given the aggressive natural history along with a high recurrence rate of uterine sarcomas, such LMS and UUS, the rationale for adjuvant chemotherapy has been repeatedly explored. In the SARCGYN phase III trial [23], adjuvant chemotherapy with cisplatin, doxorubicin, and ifosfamide followed by radiation was compared with radiation alone; although the study closed prematurely after randomizing only 81 patients, a modest improvement in 3-year DFS was observed in the chemotherapy arm (55% vs 41%; *p*=0.048), without an impact on OS. Similarly, the NRG/GOG-0277 trial, which tested adjuvant gemcitabine–docetaxel followed by doxorubicin in completely resected stage I leiomyosarcoma, was closed early for poor accrual and did not demonstrate a survival benefit compared with observation [24]. Consequently, both GEIS 2023 and ESGO 2024 guidelines do not recommend routine use of adjuvant chemotherapy in early-stage uterine sarcomas [25,26]. Nevertheless, many European centres still consider anthracycline–based regimens in selected high-risk patients, extrapolating from soft tissue sarcoma data [14,25]. According to the 2023 ESMO–EURACAN–GENTURIS soft tissue sarcoma guidelines [26], HG-ESS, adenosarcoma with sarcomatous overgrowth, and UUS are classified as high-grade tumours; while no prospective evidence supports the value of adjuvant chemotherapy, their unfavourable biology may justify individualized decision-making, especially in UUS [27].

Given the high-grade biology and elevated recurrence risk of undifferentiated uterine sarcoma (UUS), individualized treatment decisions are often made in multidisciplinary team (MDT) settings. Based on this reasoning, and supported by the soft tissue sarcoma paradigm, adjuvant anthracycline–ifosfamide chemotherapy was administered in our case. While causality cannot be inferred, this approach may plausibly have contributed to the prolonged disease-free interval observed.

Importantly, organ-intact resection without morcellation remains a fundamental oncologic principle, as it clearly minimizes the risk of peritoneal dissemination and local recurrence.

### Management of oligometastatic disease

Aggressive uterine sarcomas typically recur systemically, most often in the lungs and liver, and widespread metastases are common [22]. However, oligometastatic disease offers an opportunity for local therapy. In soft-tissue sarcomas, pulmonary metastasectomy has been associated with prolonged survival in carefully selected patients, with data from MSKCC showing a median survival of 33 months after complete resection, compared with only 11 months for patients treated nonoperatively [28]. In line with ESTRO–ASTRO definitions, our patient exhibited a genuine oligometastatic pattern (lung-only, low-volume disease) amenable to metastasis-directed interventions; SBRT series report ~80–90% 2-year local control for sarcoma lung metastases with low toxicity [10,11,29].

In our case, the multidisciplinary team (MDT) carefully balanced surgical versus non-surgical options, considering factors such as the number and location of nodules, correlation with the HRCT findings, and the interval since primary treatment. Given the limited number of well-defined pulmonary lesions and their peripheral localization, a thoracoscopic wedge resection (VATS) was deemed feasible, followed by SBRT for unresected nodules. This individualized, metastasis-directed approach achieved durable control of pulmonary relapse without significant morbidity.

Although most available evidence derives from leiomyosarcoma- or mixed-sarcoma-dominated cohorts, reports in undifferentiated uterine sarcoma (UUS) remain scarce. Our case thus contributes to bridging this knowledge gap, by virtue of illustrating that selected UUS patients with a limited metastatic burden may benefit from local ablative therapies within an MDT-guided framework. This case supports considering metastasis-directed strategies in highly selected UUS patients, particularly when managed through a multidisciplinary pathway.

### Associations with other malignancies

In cases where UUS responds to treatment, and if durable remission is achieved, patients enter long-term surveillance to monitor for recurrence and late complications. Epidemiologic data suggest that women with uterine sarcomas have an increased risk of developing second primary malignancies. A population-based Finnish study demonstrated elevated risks of breast, colorectal, and ovarian cancers in women with endometrial carcinoma and sarcoma [29]. Whether these associations reflect treatment-related effects, shared etiologic factors, or underlying tumour biology remains unclear. Given these potential risks, extended follow-up and comprehensive survivorship care are warranted.

### Limitations

This case report has several important limitations that warrant explicit discussion. First, the diagnosis of undifferentiated uterine sarcoma was established in 2016, when tumour classification was based primarily on histomorphology and immunohistochemistry according to the then-contemporaneous WHO 4^th^ edition criteria. At that time, routine molecular testing to exclude genetically defined sarcoma subtypes was not required for diagnostic assignment. Under current WHO 5^th^ edition standards, undifferentiated uterine sarcoma is regarded as a diagnosis of exclusion that ideally requires systematic molecular profiling, and this represents an inherent limitation when interpreting historical cases such as this one.

Specifically, molecular testing to exclude fusion-driven sarcomas, including high-grade endometrial stromal sarcoma (e.g., YWHAE–NUTM2 or BCOR-altered tumours), NTRK-rearranged sarcoma, PEComa, or other rare pleomorphic mimics, was not performed, and archival tissue is not available for retrospective analysis. Consequently, although the diagnosis was made according to accepted standards of the time, contemporary molecular reclassification cannot be fully excluded.

From a prognostic standpoint, this tumour exhibited several adverse features, including large size, marked pleomorphism, tumour necrosis, and absence of oestrogen and progesterone receptor expression, the latter being associated with inferior outcomes in uterine sarcomas. This biological heterogeneity may partly explain the discordance between aggressive histologic appearance and the unusually indolent oligometastatic clinical course observed.

Finally, as with all single-patient case reports, no generalizable conclusions regarding prognosis or treatment efficacy can be drawn. The patient’s prolonged survival should therefore be interpreted cautiously, but it nevertheless illustrates that selected patients with metastatic undifferentiated uterine sarcoma may achieve durable disease control with metastasis-directed therapy, even in the absence of favourable hormone receptor status.

## Conclusion

UUS is a rare and highly lethal malignancy with very limited therapeutic evidence. Prognosis remains poor in most reported series [14–16]. Nevertheless, our patient’s 8.5-year survival illustrates that an early diagnosis, expert pathological review, individualized adjuvant chemotherapy, multidisciplinary team, and metastasis-directed local therapies such as surgery and SBRT can achieve outcomes well beyond typical expectations. Collectively, these observations emphasize that long-term survivors of UUS require comprehensive survivorship plans addressing not only recurrence but also late treatment effects and the possibility of second primaries.
